# Genome-Wide Identification and Characterization of the *Cystatin* Gene Family in Bread Wheat (*Triticum aestivum* L.)

**DOI:** 10.3390/ijms221910264

**Published:** 2021-09-23

**Authors:** Long He, Xuan Chen, Miaoze Xu, Tingting Liu, Tianye Zhang, Juan Li, Jian Yang, Jianping Chen, Kaili Zhong

**Affiliations:** State Key Laboratory for Quality and Safety of Agro-Products, Institute of Plant Virology, Ningbo University, Ningbo 315211, China; hnndhelong2@163.com (L.H.); vivianccx@163.com (X.C.); xumiaoze@yeah.net (M.X.); anatkh6@163.com (T.L.); ZTye1995@163.com (T.Z.); 11716061@zju.edu.cn (J.L.); nather2008@163.com (J.Y.)

**Keywords:** *Triticum aestivum*, wheat, cystatin, gene family, gene duplication, biotic and abiotic stress

## Abstract

Cystatins, as reversible inhibitors of papain-like and legumain proteases, have been identified in several plant species. Although the *cystatin* family plays crucial roles in plant development and defense responses to various stresses, this family in wheat (*Triticum aestivum* L.) is still poorly understood. In this study, 55 wheat cystatins (*TaCystatins*) were identified. All *TaCystatins* were divided into three groups and both the conserved gene structures and peptide motifs were relatively conserved within each group. Homoeolog analysis suggested that both homoeolog retention percentage and gene duplications contributed to the abundance of the *TaCystatin* family. Analysis of duplication events confirmed that segmental duplications played an important role in the duplication patterns. The results of codon usage pattern analysis showed that *TaCystatins* had evident codon usage bias, which was mainly affected by mutation pressure. *TaCystatins* may be regulated by cis-acting elements, especially abscisic acid and methyl jasmonate responsive elements. In addition, the expression of all selected *TaCystatins* was significantly changed following viral infection and cold stress, suggesting potential roles in response to biotic and abiotic challenges. Overall, our work provides new insights into *TaCystatins* during wheat evolution and will help further research to decipher the roles of *TaCystatins* under diverse stress conditions.

## 1. Introduction

Protein hydrolysis in eukaryotic cells is a complex and sophisticated process that is regulated by a series of endogenous or exogenous proteases [1,2]. Proteases can be divided into different families according to their amino acid residues at their reaction sites [3]. Among these proteases, the family C1A proteases, namely papain-like cysteine proteases [PLCPs], and the family C13 proteases, namely legumain-like cysteine proteases [LLCPs], play important roles in various physiological processes [4,5,6,7]. The protease activity of PLCPs and LLCPs is affected by a group of small proteins called cystatins [8]. Cystatins have been reported to be tight and reversible inhibitors of C1A and C13 proteinases in multiple plants [9].

The *cystatin* family has been identified and characterized in pests, mammals, and plants [10,11,12]. All identified cystatins have three typical conserved motifs, which include a QxVxG motif in the reaction site, glycine residues in the N-terminus, and one tryptophan residue in the C-terminus [13]. These three conserved motifs can directly bind to the active center of the cysteine protease, resulting in the inhibition of catalytic activity [6,14]. In addition, a consensus sequence, namely (LVI)-(AGT)-(RKE)-(FY)-(AS)-(VI)-x-(EDQV), was found to be general for all cystatins identified in plants, which was related to a predicted secondary α-helix structure [15]. Compared to animal cystatins, plant cystatins are a group of proteins with a molecular weight (MW) ranging from 12 to 16 kDa, lacking glycosylation sites and disulfide bonds [6,16]. Several plant cystatins are usually thought to be special inhibitors of LLCPs due to an extended C-terminus which allows their MW to reach 23 kDa [15,17].

The primary function of plant cystatins is the regulation of cysteine proteases, which are involved in various physiological processes including plant growth and development, senescence, seed development and germination, nitrogen fixation, sexual reproduction, embryogenesis, and programmed cell death (PCD) [14,18,19,20,21,22]. More importantly, plant cystatins have been reported to participate in the regulation of plant defense against abiotic or biotic stress, including pathogens, pest attack, heat stress, and exogenous hormone treatments. For example, cystatins have been described to prevent attacks by mites and pathogenic fungi [23,24]. Application of exogenous methyl jasmonate (MeJA) positively modulates plant defense through the induction of cystatin expression against *Tilletia indica* infection, which causes serious losses to grain yields [25]. Karina et al. cloned a *cystatin* gene in maize called *CC9*, which inhibited the plant host immunity response by affecting apoplastic cysteine proteases [22]. A previous report has shown that the application of exogenous abscisic acid (ABA) or heat stress treatments can lead to the induction of *AtCYS5* expression and further investigation revealed that the transgenic *Arabidopsis* lines overexpressing AtCYS5 display enhanced resistance against heat threats [26]. Tan et al. observed that overexpression of MpCYS2, a *cystatin* gene cloned from *Malus prunifolia* (Willd.) *Borkh* in *A. thaliana*, dramatically enhances drought tolerance [27].

Bread wheat (*Triticum aestivum*) is the most widely cultivated grain worldwide and supplies approximately one-fifth of the total calories consumed by humans [28]. With the growth of the world’s population, it is predicted that wheat agricultural production needs to be increased by 38% to satisfy the growing demand for food [29]. The cystatin family has been well characterized in a number of plant species such as *A. thaliana*, soybean, apple, and a variety of cereal crops, including rice, *Brachypodium distachyon,* and sorghum [10,11,12,30,31,32,33]. However, little is known about the *cystatin* family in bread wheat. As a complex allohexaploid with a large number of repetitive and transposable elements, bread wheat has one of the largest crop plant genomes (16 Gb genome size; AABBDD genomes), which makes working with bread wheat challenging from a genetics, genomics, and breeding perspective [34]. Fortunately, with the rapid development of genome sequencing technology, a high-quality complete genome assembly and annotation of wheat organized by the International Wheat Genome Sequencing Consortium have been completed, which provides us with a good opportunity to identify and characterize *cystatin* family members in wheat [35].

In the present study, a genome-wide investigation of the *cystatin* family in wheat was performed based on the recently released genome of *T. aestivum* [35]. We identified and characterized 55 members of the cystatin gene family in wheat bread. Furthermore, an overview of gene structures, evolutionary relationships, expansion, and expression levels of the wheat genes from the *cystatin* gene family is provided. In summary, our work provides a novel viewpoint for the subsequent research into *cystatin* genes in bread wheat and may contribute to further functional studies of *cystatin* genes to enhance the resistance of bread wheat against various stresses.

## 2. Results

### 2.1. Genome-Wide Identification of the TaCystatin Family

Cystatins were identified and characterized in *Arabidopsis thaliana* and rice (*Oryza sativa)* (10,30), and their locus IDs and sequences are listed in Supplementary Table S1. To extract all cystatin members in bread wheat (*Triticum aestivum)*, we performed a genome-wide analysis through local BLSATP using *A. thaliana* and rice cystatin protein sequences as queries. All candidates were further filtered using Protein family database (Pfam) search and NCBI Batch CDD for function annotations. In this study, we identified 55 cystatins in wheat. The gene ID, location and open reading frame (ORF) length, amino acid length, MW, isoelectric points (PIs), gravy, and subcellular location are listed in Table 1. The ORF of *TaCystatins* ranged from 318 (*TraesCS3D02G416600.1*) to 546 (*TraesCS1A02G2564000.1*) bp in length. The lengths of TaCystatin proteins ranged from 100 (TraesCS2A02G576200.1) to 243 (TraesCS3B02G215400.1) amino acid residues, MW ranged from 11.5 to 26.7 kDa, and the PI ranged from 5.01 (TraesCS5D02G502100.1) to 10.23 (TraesCS2D02G274900.1). In addition, analysis of the subcellular localization showed that more than half of the identified TaCystatins were localized in the extracellular space, while only 10.9%, 26.7%, and 20.0% of the identified TaCystatins were predicted to be localized in the chloroplast, mitochondria, and cytoplasm, respectively.

### 2.2. Phylogenetic and Classification Analysis of Cystatins

**Figure 1:**
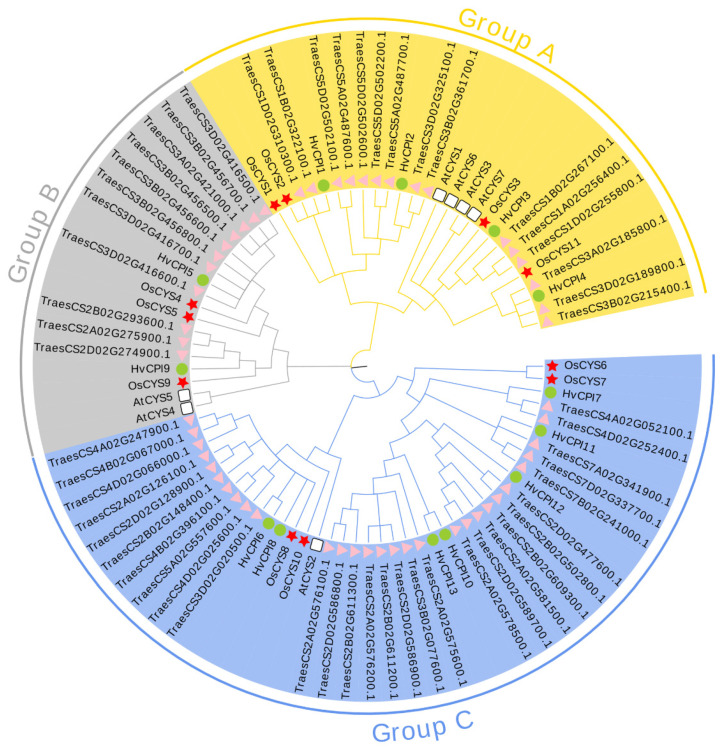
Phylogenetic tree of cystatins. The maximum likelihood (ML) tree was constructed on the basis of the full-length amino acid sequences of bread wheat (55), *Arabidopsis thaliana* (7), rice (11), and barley (13) using MEGA X software, with a set of 1000 replications. All cystatins were divided into three phylogenetic groups, and each group is represented by a different color. Triangles, stars, circles, and squares correspondingly indicate bread wheat, rice, barley, and *A. thaliana*.

**Figure 2:**
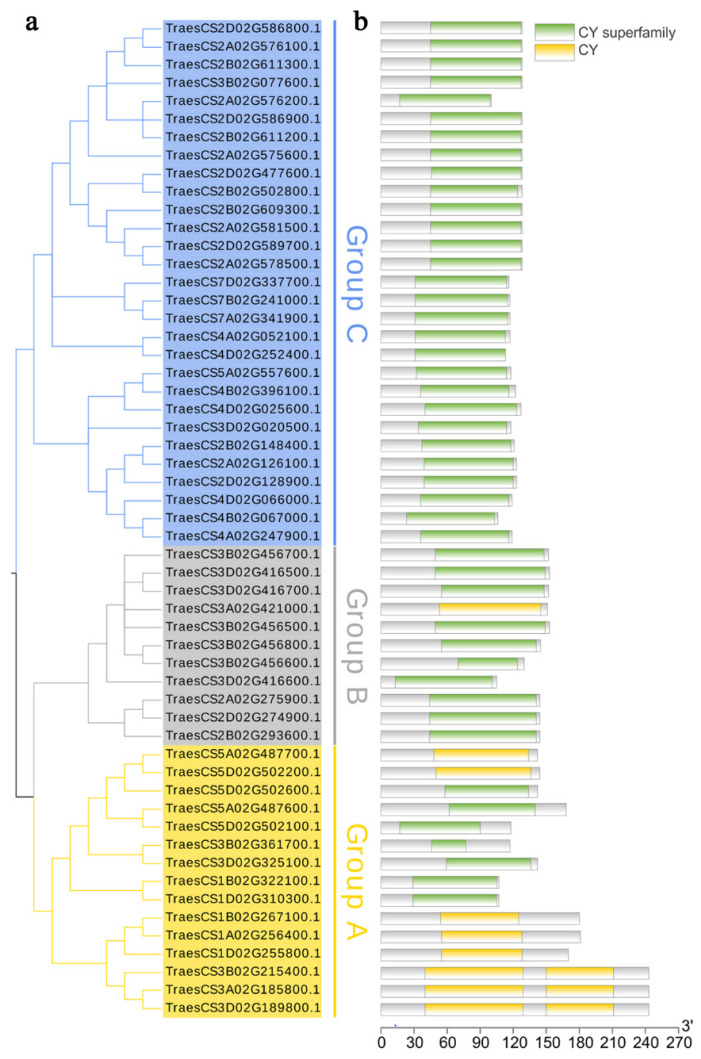
Phylogenetic and conserved domain analysis of the *TaCystatin* family. (**a**) Phylogenetic tree of TaCystatins. The ML tree was constructed on the basis of the full-length amino acid sequences of TaCystatins by MEGA X, with a set of 1000 replications. All TaCystatins were divided into three phylogenetic groups. (**b**) Conserved domain of 55 TaCystatins.

To explore the phylogenetic relationships between TaCystatins and other known plant cystatins, seven *A. thaliana*, 11 rice, 13 barley, and 55 wheat cystatin protein sequences were used to construct a maximum likelihood phylogenetic tree using MEGA X (Figure 1). Similarly, we also constructed a maximum likelihood phylogenetic tree using only 55 wheat cystatin protein sequences (Figure 2a). Using the NCBI Batch CDD tools, we confirmed that all the identified TaCystatin sequences had one or two conserved cystatin domains (Figure 2b). All identified TaCystatins were clustered into three groups: Group A, Group B, and Group C, which was consistent with previous reports [6,11]. In addition, we found that wheat shows both a greater number of cystatin genes than the other analyzed plants and a greater abundance of all clades (Table 2).

### 2.3. Duplication Events Analysis of the TaCystatin Family

The *cystatin* family has been identified and characterized in several flowering plants (12, 15, 30). We found that the number of cystatin genes in bread wheat was the highest among the analyzed plants (Table 2). To better understand why the number of *cystatin* genes in wheat was quite high, we analyzed the homoeologous groups for the *TaCystatin* family. In this study, the results showed that 43.65% of the *TaCystatin* genes displayed in homoeologous groups (1:1:1), representing that three *TaCystatins* localized on the B, D, and A sub-genome shared high homology, which were also defined as triads (Table 3). Previous studies have reported that 35.8% of wheat genes are present in triads [36,37]. The proportion of homoeologous triads of the *cystatin* family was higher than that in the whole wheat genome (43.65% vs. 35.8%; Table 3). Hence, the higher proportion suggests that the expansion of *TaCystatin* may result from wheat polyploidization. However, even with respect to the ploidy level caused by the fact that wheat exhibits complex hexaploidy, the number of cystatin genes in wheat was still much higher than that in rice (Figure S1), which were the model species of monocots. In addition, the proportion of homoeologous groups with gene duplications in one sub-genome (1:1: N, N:1:1, or 1: N:1) was dramatically higher than that in the whole wheat genome (14.55% vs. 5.7%; Table 3), while the proportion of homoeologous groups in which there was one homoeolog (1:1:0, 0:1:1, or 1:0:1) was quite close to that of the whole wheat genome (14.54% vs. 13.2%; Table 3). Thus, our results suggest that the higher number of *TaCystatin* genes is partly due to the homoeolog retention percentage and gene duplications.

**Figure 3:**
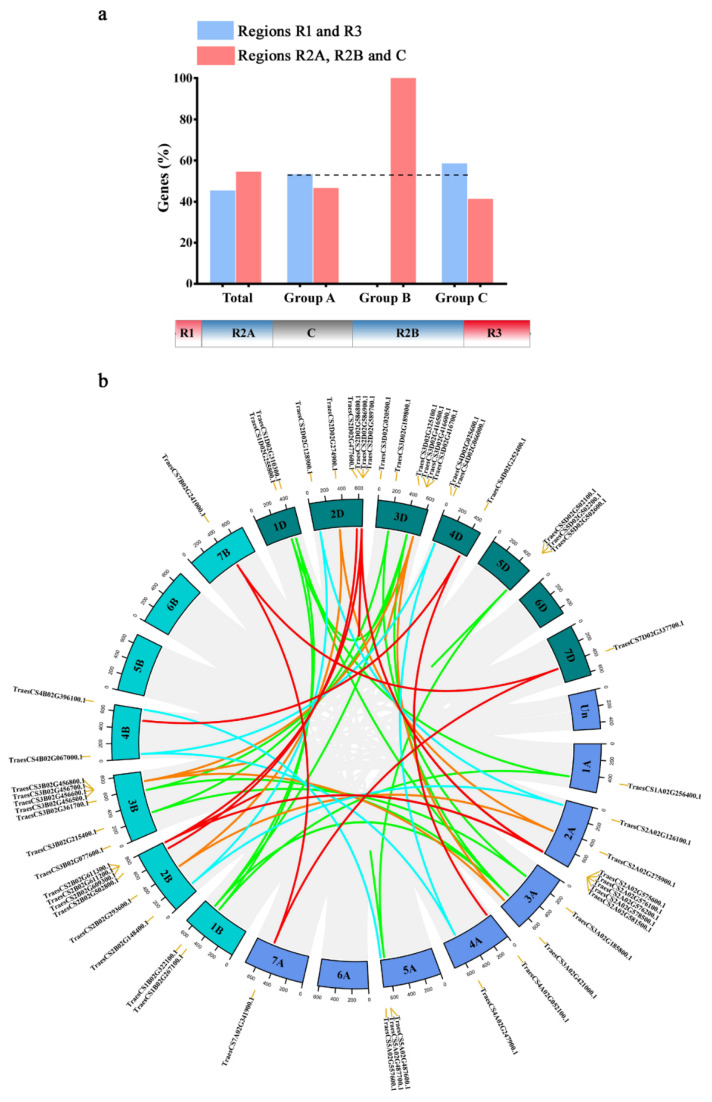
Chromosomal distribution of the *TaCystatin* family. (**a**) The ratio between the *TaCystatin* genes in R2A, R2B together with C segments of every chromosome, and R1 together with R3 segments of every chromosome. (**b**) Genomic location and duplication events analysis of 55 *TaCystatin* genes. Light-gray lines in the background indicate the synteny blocks within the bread wheat genome. The duplication events are highlighted with different colored lines.

To better investigate the features of the *TaCystatin* family, we conducted chromosomal location analysis. The wheat A, B, and D sub-genomes contained 16, 18, and 21 *cystatin* genes, respectively (Figure S2). The wheat chromosome 2 contained the largest number of *cystatin* genes, with seven *cystatin* genes on chromosome 2A, six *cystatin* genes on chromosome 2B, and six *cystatin* genes on chromosome 2D. No cystatin gene family members were identified on chromosomes 6A, 6B, 6D, and 5B. Chromosomal location analysis revealed that the *TaCystatins* were unevenly distributed on chromosomes. We further analyzed the distribution of *TaCystatin* genes within each chromosome. Detailed information is provided in Table S2 and is illustrated in Figure 3a. The results showed that the proportion of *TaCystatin* distribution in the distal telomeric regions of the chromosomes and the proportion of *TaCystatin* distribution in the more central regions of the chromosomes were similar (47.45% vs. 54.55%; Table S2). Interestingly, we found that the proportion of the *TaCystatin* distribution in distal and proximal chromosomal regions varied greatly among Group A (53.33% vs. 46.67%), Group B (0 vs. 100.0%), and Group C (58.62% vs. 41.38%), which corresponded to the number of members of their phylogenetic group. Overall, the *TaCystatins* belonging to the greater phylogenetic group were inclined to be located in distal telomeric regions of the chromosomes, consistent with previous findings [37]. Tandem and segmental duplications have been recognized as two main factors in the expansion of gene families in plants [38]. Here, we identified eight tandem duplication clusters in the *TaCystatin* family (Figure 3b). Three tandem duplications occurred on chromosome 3B and the remaining tandem duplications occurred on chromosomes 2B, 2D, 3D, 5A, and 5D. In addition, 45 collinear *cystatin* gene pairs (Figure 3b) were characterized, which indicated that segmental duplications occupied a significant position in the expansion of the *TaCystatin* family.

### 2.4. Analysis of Cystatin Paralogs and Orthologs

**Figure 4:**
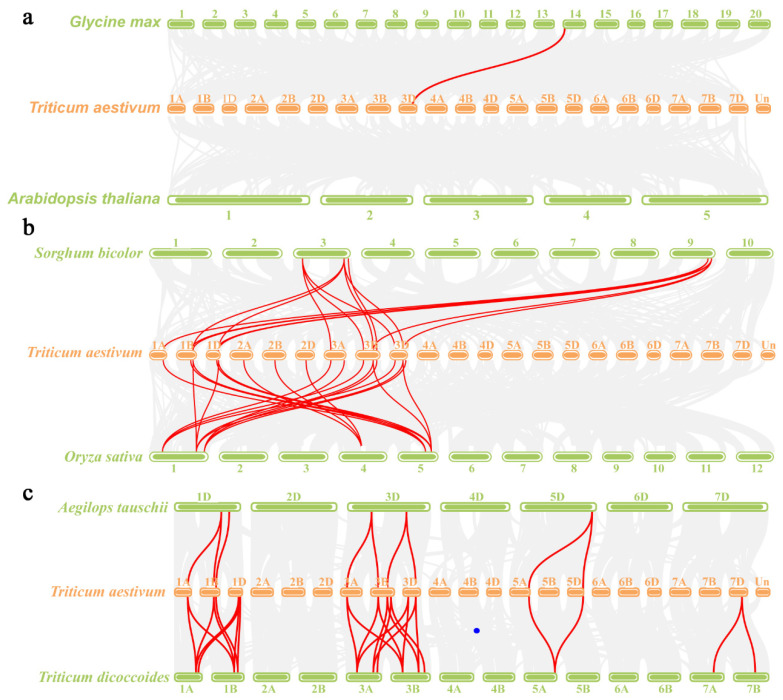
Syntenic relationships of the *cystatin* genes in bread wheat and six other species. (**a**) Syntenic relationships of *cystatins* between wheat, *Arabidopsis thaliana*, and *Glycine max*. (**b**) Syntenic relationships of *cystatins* between wheat, *Oryza sativa*, and *S.bicolor.* (**c**) Syntenic relationships of *cystatins* between wheat, *Aegilops tauschii*, and *Triticum dicoccoides.* Gray lines in the background represent the synteny blocks of wheat and other plants, while the red lines highlight the syntenic *cystatin* gene pairs.

To further investigate the evolutionary relationships of the *cystatin* family, we conducted syntenic analysis using the McscanX software to identify *cystatin* paralog gene pairs in wheat (*T. aestivum*) and *cystatin* orthologous gene pairs between wheat and dicotyledonous plants (*Arabidopsis* and *G. max*), monocotyledonous plants (*O. sativa* and *Sorghum bicolor*), and wheat relatives (*Aegilops tauschii* and *Triticum dicoccoides*). In this study, we identified 45 paralogues in bread wheat (*Ta*-*Ta*) (Table S3). No *cystatin* orthologous gene pairs were observed between *Arabidopsis* and wheat (*Ta*-*At*), and only one *cystatin* orthologous gene pair was found between *G. max* and wheat (*Ta*-*Gm*) (Figure 4 and Table S4). We also found that 10 and 15 *TaCystatin* genes were collinear with *cystatin* genes in rice (*O. sativa*) and *Sorghum bicolor* (*Ta*-*Os* and *Ta*-*Sb*), respectively (Figure 4 and Table S4). Eleven and twenty-five orthologous *cystatin* gene pairs were identified in wheat with *Aegilops tauschii* and *Triticum dicoccoides* (*Ta*-*Aet* and *Ta*-*Td*), respectively. These results suggest that *cystatin* genes in wheat are distantly related to those in dicotyledonous species and are most closely associated with those in *T. dicoccoides*, which might be due to the fact that *T. dicoccoides* are widely considered to be A-genome and B-genome donors of wheat [39]. The *Ka/Ks* ratio indicated the selective pressure on plant genes, which can be used to diagnose the evolutional form of the sequence [37]. The divergence time (T) was calculated according to *Ks* values. We estimated the *Ka, Ks*, *Ka*/*Ks*, and T values for each cystatin gene pair to further decipher the evolutionary trends of the *cystatin* family. In general, Ka/Ks > 1 represents accelerated evolution with advantageous selection, Ka/Ks = 1 represents neutral selection, and Ka/Ks < 1 represents purifying selection [38]. We found that the Ka/Ks ratios of most paralogous genes (*Ta*-*Ta*) and all orthologous genes (*Ta*-*Gm*, *Ta*-*Os*, *Ta*-*Sb*, *Ta*-*Aet,* and *Ta*-*Td*) were less than 1 (Tables S3 and S4), suggesting that purifying selection plays a more significant role during the evolution of the *cystatin* family. In addition, the results showed that the divergence time of 45 paralogues (*Ta*-*Ta*) ranged from 0.691149 to 39.22863 Mya. The divergence time of orthologues (*Ta*-*Gm*, *Ta*-*Os*, *Ta*-*Sb*, *Ta*-*Aet,* and *Ta*-*Td*) varied greatly depending on species and the divergence time of orthologues (*Ta*-*Gm*) was the longest, while *Ta*-*Td* was the shortest (Table S4).

### 2.5. Codon Usage Pattern Analysis of Cystatin Genes in Plants

**Figure 5:**
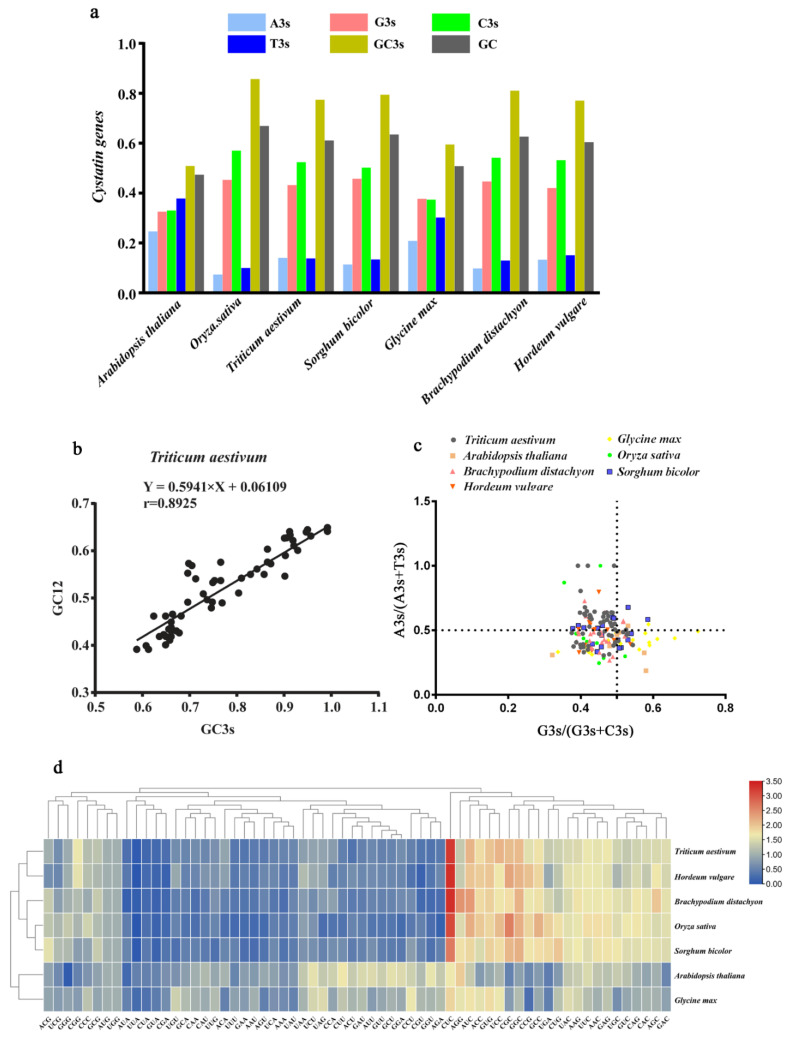
Codon usage pattern analysis. (**a**) Vertical axis representing the contents of various bases at the third position of the codons in seven species. (**b**) Neutrality plot analysis of TaCystatin CDS sequences. (**c**) Parity rule 2 (PR2) analysis of cystatin CDS sequences in seven species. (**d**) Heatmap showing relative synonymous codon usage (RSCU) values of cystatin CDS sequences in seven species. Blue-to-red color indicates low-to-high RSCU values of codons.

Triplet codons play an important role in biological information transmission. Each codon encodes one amino acid, while an amino acid corresponds to at least one and up to six codons, among which the multiple codons encoding the same amino acid are called synonymous codons [40,41]. However, the frequency of synonymous codon usage varies greatly among different species or genes [42]. The codon preference of synonymous codon usage is an important parameter of evolutionary processes. CUB is also important for the level of gene expression, as it affects the translation efficiency and functional differentiation of genes [43,44]. In general, the GC content of the third codon position (GC3) is widely considered to be related to codon usage patterns [45]. Codon usage patterns of *cystatin* genes in seven species were analyzed using each *cystatin* CDS sequence and detailed information on codon usage indicators is provided in Table S5. We observed that the average GC proportion of *cystatin* genes was lower in dicot species than in monocot species (Figure 5a). The results also showed that the average proportion of A/T-terminated codons of *cystatin* genes in dicot species was relatively higher, while G/C-terminated codons were more common in monocot species (Figure 5a), which was consistent with previous reports [46,47]. The average GC3s and GC content of *cystatin* genes in rice (*O. sativa*) were higher than those in other species (Table 4). Compared with the *cystatin* genes in other species, the average effective number of codons (ENC) in rice was the lowest (Table 4), followed by *B. distachyon* and bread wheat (*T. aestivum*). This suggests that the CUB of *cystatin* genes in rice was the strongest, followed by *B. distachyon* and bread wheat. It is widely accepted that relative synonymous codon usage (RSCU) can intuitively indicate CUB [48]. RSCU > 1 represents more used codons, while RSCU < 1 means that codons are used less frequently than expected, and RSCU = 1 indicates that codons have no preference [49]. Thus, we performed relative (RSCU) analysis to better understand the codon usage patterns of *cystatin* genes in seven plant species. We found that the RSCU values of *cystatin* genes were close in two dicotyledons, namely *A. thaliana* and *G.max*, and the RSCU values of that in five monocots were also relatively similar (Figure 5d). Among the five monocotyledon plants, the RSCU values of *cystatin* genes in common wheat and barley (*H. vulgare*) were clustered in one group, while those in rice and *Sorghum bicolor* were clustered in the other (Figure 5d), which might be related to evolutionary relations between these species.

We performed parity rule 2 (PR2) analysis to investigate the bias of the AT and GC composition. We found that there were differences between T, A, G, and C proportions in most *cystatin* genes (Figure 5c and Figure S3). The results showed that C and A were used more frequently than G and T in common wheat. C and T were used more frequently than G and A in other species except for G3s/(G3s+C3s) in *G. max*, which suggests that mutation pressure might work in the nucleotide composition. We performed neutral plot analysis to further confirm the presence of other factors for codon preference, except for the base composition. A positive and significant correlation between GC3s and GC12 was detected in the *cystatin* gene CDS sequences of common wheat (r = 0.8925, *p* < 0.01) (Figure 5b). The GC3s of *cystatin* gene CDS sequences in common wheat ranged from 0.588 to 0.992. The slope of the regression line for *cystatin* genes in common wheat was 0.5941, which suggests that the effect of mutation pressure on the codon preference of *cystatin* genes in wheat was 59.41%. Neutral plot analysis was also performed for *cystatin* genes in the six other species. As shown in Figure S4, a positive and significant correlation between GC3s and GC12 was detected in *cystatin* gene CDS sequences of barley (r = 0.5800, *p* < 0.01), *B. distachyon* (r = 0.6667, *p* < 0.01), *S. bicolor* (r = 0.6244, *p* < 0.01), and *G. max* (r = 0.8032, *p* < 0.01). In addition, we found that the slope values of the regression line for *cystatin* genes in *A. thaliana* and *G. max* were lower than those in the rest of the analyzed species, which might be due to the fact that both *A. thaliana* and *G. max* are dicotyledons. In summary, our results suggest that the codon usage pattern of *cystatin* genes is relatively conserved within dicotyledonous or monocotyledonous plants and the CUB of *cystatin* genes in monocots was relatively stronger than that in dicots, consistent with previous findings [46].

### 2.6. Conserved Motifs and Gene Structure Analysis of TaCystatin

**Figure 6:**
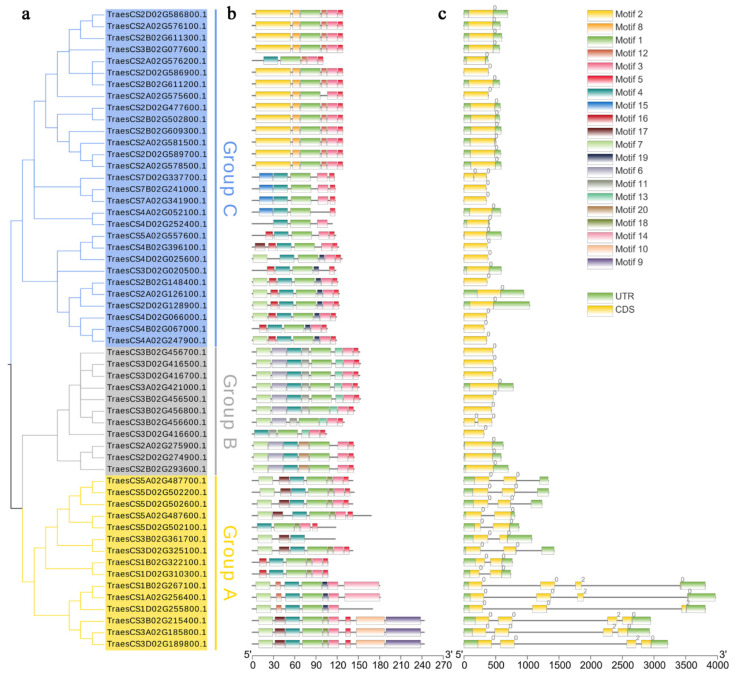
Phylogenetic relationship, gene structure, and motif composition analysis of TaCystatins. (**a**) Phylogenetic tree of TaCystatins. The ML tree was built on the basis of the full-length amino acid sequences of TaCystatins using MEGA X, with a set of 1000 replications. All TaCystatins members were divided into three phylogenetic groups. (**b**) Architecture of the conserved protein motifs of TaCystatins. Different motifs numbered 1 to 20 are indicated by different colors. (**c**) Intron/exon structures analysis of *TaCystatins*. Untranslated regions (UTRs) are represented by green boxes, introns are represented by gray lines, and the coding sequences (CDS) of exons are indicated by yellow boxes.

The comparison of the gene exon–intron structure provides novel insights into the evolution and function of gene family members [12]. To investigate the structural features of *TaCysatin* genes, we analyzed the exon–intron distribution of *TaCysatins* using TBtools software [50]. The results showed that the number of introns of *TaCysatin* genes ranged from one to four (Figure 6c). Most members of the *TaCysatin* genes from Group C or B had only one intron, while all members from Group A had the least number of two introns (Figure 6c). Overall, the exon–intron structures of most genes within the same group were relatively conserved. To characterize the conserved motifs of the identified TaCystatins, we submitted the amino acid sequences of all TaCystatin proteins to the MEME online website. Twenty conserved motifs were predicted, as illustrated in Figure 6b. The results revealed that the motif number of TaCystatins varied from three to nine. Several motifs were prevalent for most TaCystains, while others only existed in certain groups. For example, 98% of TaCystain members contained motif 1, while 87% of TaCystain members contained both motifs 3 and 5. Motif 2 was unique to Group C. Motifs 6 and 11 only existed in Group B. In summary, a number of TaCystatins within the same group shared similar motif structures and the genes of the same motifs’ composition might have similar functions. Detailed information on the 20 conserved motifs is provided in Figure S5.

### 2.7. Prediction of Cis-Acting Regulatory Elements in Promoter Regions of TaCystatins

**Figure 7:**
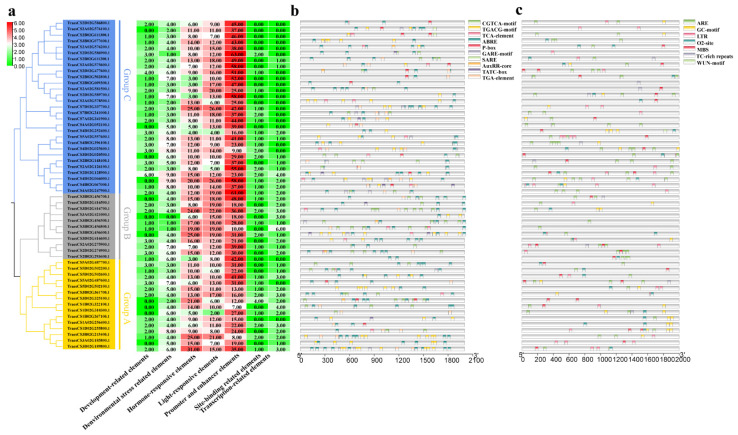
Prediction analysis of cis-acting regulatory elements in *TaCystatins*. (**a**) The number of seven different kinds of cis-acting elements in the promoter region of *TaCystatins*. (**b**) The type and position of hormone-responsive elements in *TaCystatins*. (**c**) The type and position of environmental stress-related elements in *TaCystatins*.

Cis-acting regulatory elements located in the promoter region can regulate the expression levels of target genes by binding to transcription factors [51,52]. Cis-acting elements have been reported to be involved in various plant responses to abiotic or biotic stress [53,54,55,56]. To explore the expression pattern of *TaCystatin* genes, we submitted the 2.0 kb promoter region sequences of *TaCystatins* to the PlantCARE database website [57]. In summary, 3647 cis-acting regulatory elements were identified. All identified cis-acting regulatory elements could be classified into several categories, including development-related, environmental stress-related, hormone-responsive, light-responsive, promoter and enhancer, site-binding-related, and transcription-related elements (Figure 7a), which suggested that cis-acting elements of *TaCystatins* play a significant role during wheat growth and development. We identified 278 environmental stress-related elements (Figure 7c). These predicted environmental stress-related elements were involved in temperature, drought, and pathogen responses. In addition, a total of 651 hormone-responsive elements were identified. ABREs responding to ABA and CGTCA-motifs responding to MeJA accounted for the majority of these predicted hormone-responsive elements. In fact, ABA-responsive cis-acting and MeJA-responsive cis-acting elements were characterized in the promoter regions of all the identified *TaCystatin* genes (Figure 7b).

**Figure 8:**
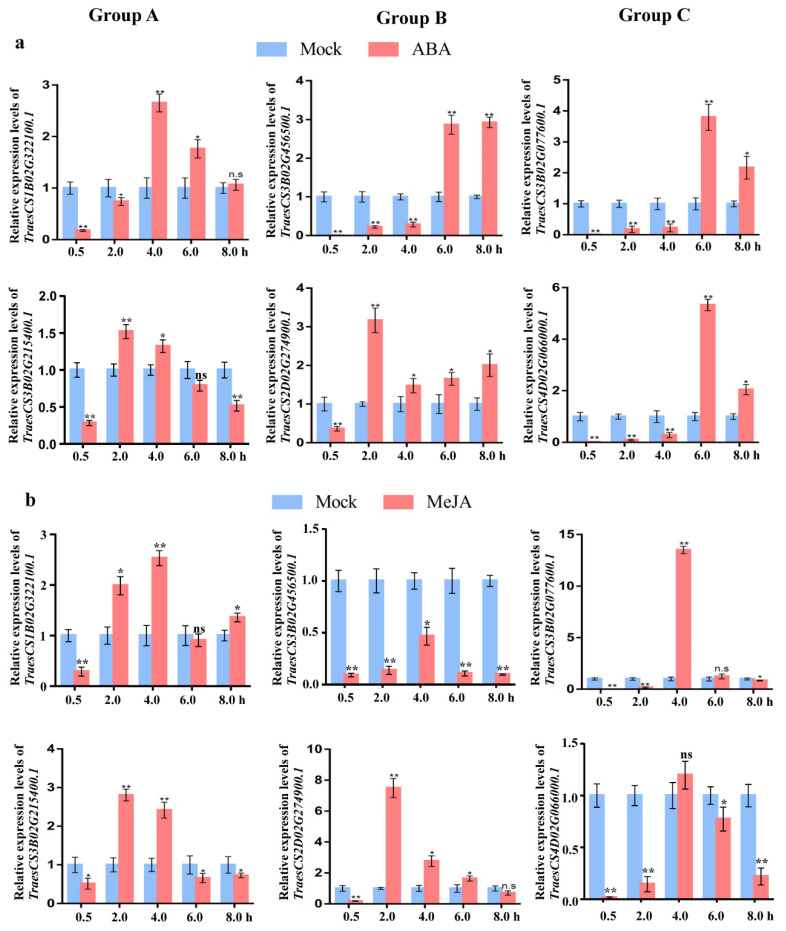
Expression pattern analysis of *TaCystatin* genes upon hormone applications. (**a**) Relative expression levels of six selected *TaCystatin* genes at 0.5, 2, 4, 6, and 8 h after 100 μmol L^−1^ ABA treatments. Means ± standard deviations (SE) were deciphered from three biological replicates and each biological replicate had three technical replicates. Note: ns, not significant; **, *p* < 0.01; and *, *p* < 0.05 (Student’s *t*-test). (**b**) Relative expression levels of six selected *TaCystatin* genes at 0.5, 2, 4, 6, and 8 h after 100 μmol L^−1^ MeJA treatments. Means ± standard deviations (SE) were deciphered from three biological replicates and each biological replicate had three technical replicates. Note: ns, not significant; **, *p* < 0.01; and *, *p* < 0.05 (Student’s *t*-test). Plants treated with 0.1% Triton X-100 were used as the mock control.

Cis-acting regulatory element analysis showed that *TaCystatin* genes were mostly characterized by ABA and MeJA. To investigate the association between the *cystatin* family in wheat and ABA or MeJA, we randomly selected two members from each phylogenetic group of the *TaCystatin* family as representatives to measure the expression profiles of six selected *TaCystatins* upon ABA or MeJA exogenous treatments by quantitative reverse transcription polymerase chain reaction (RT-qPCR). The results showed that all selected *TaCystatin* genes were sensitive to ABA or MeJA application (Figure 8), which, to some extent, indicated a close relationship between the selected *TaCystatin* genes’ regulation and ABA together with MeJA. Both ABA and MeJA have been shown to play important roles in plant stress biology [58,59,60,61]. Our results suggest that the expression of *TaCystatin* genes may be involved in several different stresses.

### 2.8. Tissue-Specific Expression Analysis of TaCystatins

**Figure 9:**
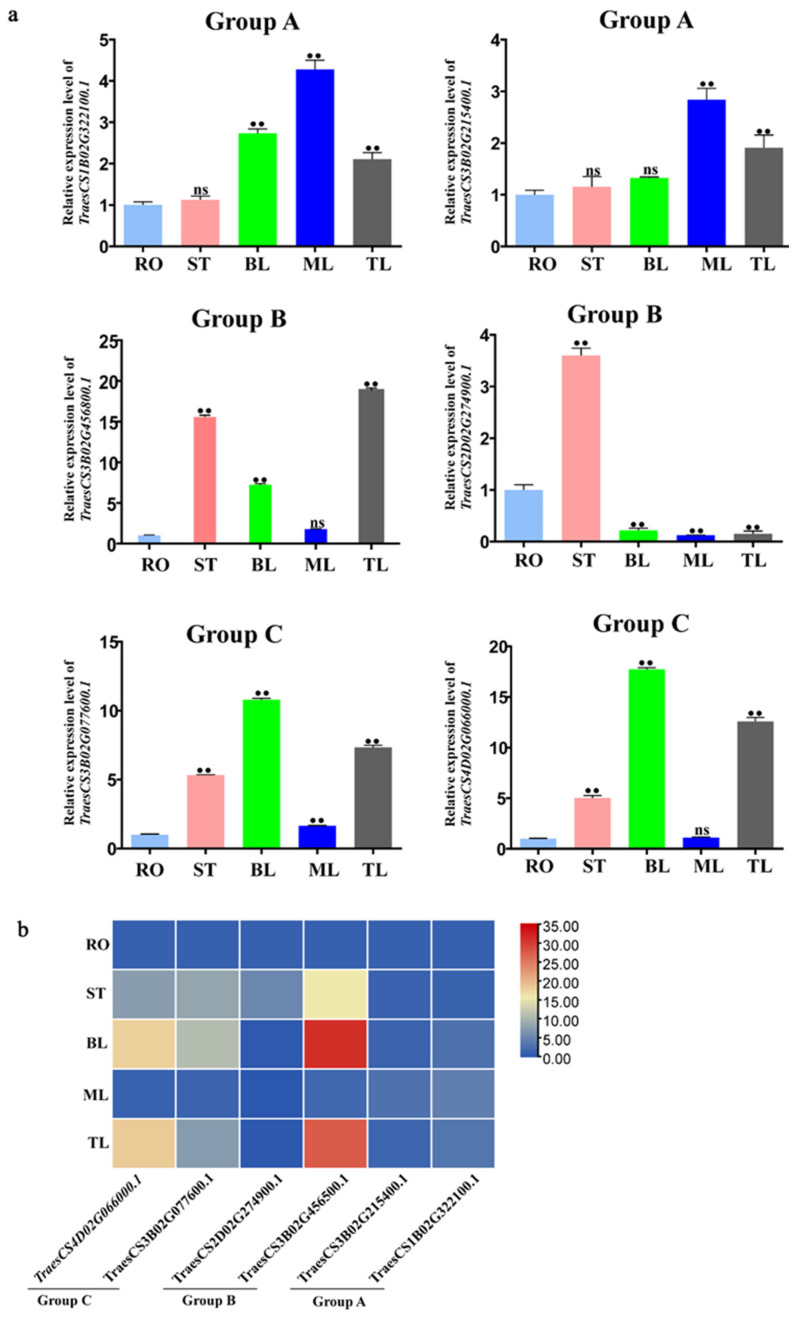
Expression pattern analysis of selected *TaCystatin* genes among different organs. (**a**) Raw data indicating the relative expression levels of selected *TaCystatin* genes in distinct organs. Means ± standard deviations (SE) were deciphered from three biological replicates and each biological replicate had three technical replicates. Note: ns, not significant and **, *p* < 0.01 (Student’s *t*-test). (**b**) Heatmap indicating the relative expression of the selected *TaCystatin* genes in distinct organs. Color scale represents relative expression values, with the color from blue to red indicating low to high expression abundance. Abbreviations: RO, roots; ST, stem; BL, bottom leaf; ML, middle leaf; and TL, top leaf. The expression of RO was regarded as the controls.

To comprehensively decipher the functions of *TaCystatin* genes, we calculated the expression levels of six selected *TaCystatin* genes in five different tissues of bread wheat by RT-qPCR. Five different organs from which we collected samples contained roots (RO), stem (ST), bottom leaf (BL), middle leaf (ML), and top leaf (TL) in three-leaf-stage bread wheat. The expression of the selected genes in RO were regarded as the mock control. As illustrated in Figure 9, all selected *TaCystatin* genes were expressed in at least one organ. Most of the selected *TaCystatin* genes showed distinct expression patterns between different phylogenetic groups. However, their expression pattern was relatively similar within the same phylogenetic group. For instance, the expression levels of all the selected genes were relatively higher in the top leaves and relatively lower in the roots (Figure 9), except for *TraesCS2D02G274900.1*.

These results suggest that various *TaCystatins* may be involved in the development of different tissues during various stages.

### 2.9. Expression Analysis of TaCystatins under Abiotic and Biotic Stress

**Figure 10:**
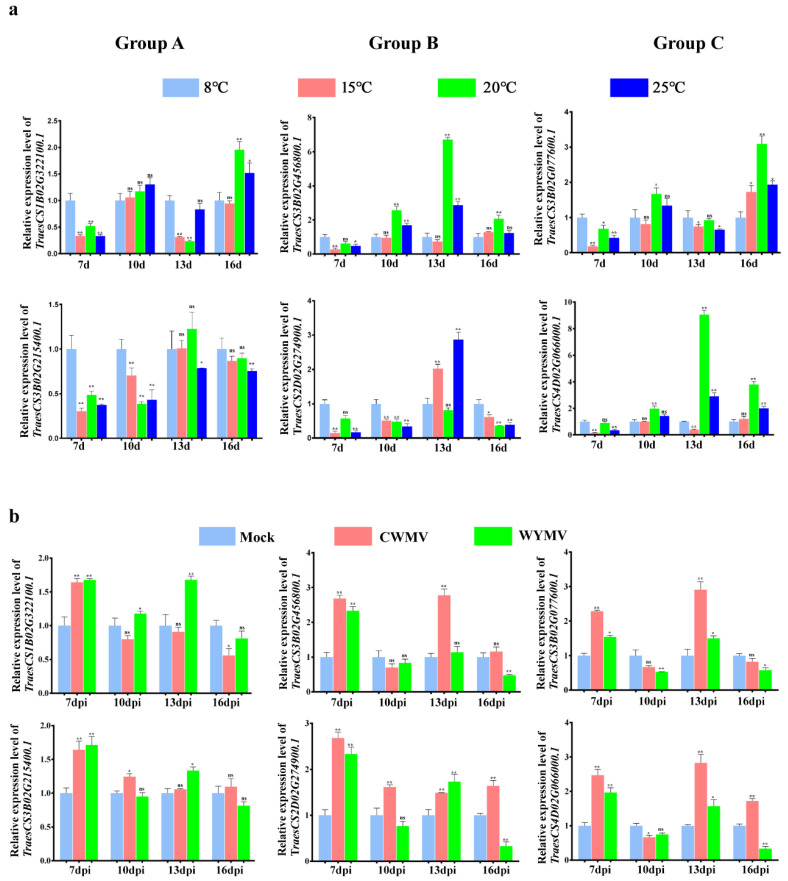
Expression analysis of selected *TaCystatins* genes under biotic or abiotic stress. (**a**) Expression profiles of selected *TaCystatins* genes under different temperatures calculated by RT-qPCR. Means ± standard deviations (SE) were deciphered from three biological replicates and each biological replicate had three technical replicates. Note: ns, not significant; **, *p* < 0.01; and *, *p* < 0.05 (Student’s *t*-test). (**b**) Expression profiles of selected *TaCystatin* genes under viral infection analyzed by RT-qPCR. Means ± standard deviations (SE) were deciphered from three biological replicates and each biological replicate had three technical replicates. Note: ns, not significant; ** *p* < 0.01; and * *p* < 0.05 (Student’s *t*-test).

Previous studies have found that plant cystatins are widely involved in plant growth and development, senescence, and PCD [20,21,22,61,62]. The expression levels of several plant *cystatin* genes have been reported to be affected by various conditions, including pathogens and cold stress [63,64]. Climate temperature is one of the most important factors affecting wheat production; the Chinese wheat mosaic virus (CWMV), as well as the wheat yellow mosaic virus (WYMV), pose a severe threat to winter wheat production in China [65,66]. Thus, to explore the potential roles of *TaCystatins* in response to biotic or abiotic stress, we analyzed the effects of viral inoculation (biotic) and gradient temperature treatment (abiotic) on the expression levels of selected *TaCystatin* genes. We found that the expression levels of all the selected genes were dramatically up-regulated at seven days post inoculation (dpi) with CWMV or WYMV (Figure 10b). The expression levels of *TraesCS1B02G322100.1*, *TraesCS3B02G456800.1*, and *TraesCS3B02G77600.1* did not change significantly at 10 days post CWMV infection, while significant changes in the expression of *TraesCS3B02G215400.1*, *TraesCS2D02G274900.1*, and *TraesCS4D02G066000.1* were observed at 10 days post CWMV infection. Regarding 10 days post WYMV infection, *TraesCS1B02G322100.1* and *TraesCS3B02G77600.1* displayed important expression changes. Meanwhile, there were no significant changes observed in the expression of *TraesCS3B02G456800.1*, *TraesCS3B02G215400.1*, *TraesCS2D02G274900.1*, and *TraesCS4D02G066000.1*. We found that the expression of *TraesCS3B02G456800.1* was dramatically upregulated at 13 days post CWMV inoculation, while its expression levels did not change significantly at 13 days post WYMV inoculation. The expression of *TraesCS1B02G322100.1* was significantly increased by WYMV but not by CWMV (Figure 10b). In the final stage of plant responses for inoculation with the virus, the expression of *TraesCS2D02G274900.1* and *TraesCS4D02G066000.1* were both up-regulated by CWMV infection, while down-regulated by WYMV infection. Moreover, we found that the expression of *TraesCS1B02G322100.1* and *TraesCS3B02G215400.1* did not respond to WYMV infection, and *TraesCS3B02G456800.1* as well as *TraesCS3B02G77600.1* did not respond to CWMV infection. Most *TaCystatin* genes were highly expressed at 8 °C on the seventh day (Figure 10a) compared to that under 15, 20, or 25 °C. Interestingly, the situation became quite complex and diverse on the 10th day. *TraesCS2D02G274900.1* showed high expression levels at 25 and 15 °C on the 13th day, while its expression was relatively weakened at 20 °C (Figure 10a). On the 16th day, we observed that the expression levels of some members including *TraesCS1B02G322100.1*, *TraesCS3B02G456800.1*, *TraesCS3B02G77600.1*, and *TraesCS4D02G066000.1* were upregulated at high temperatures (20 and 25 °C) (Figure 10a). Additionally, *TraesCS3B02G215400.1* showed relatively low expression levels at all time points at high temperatures. Interestingly, we also observed that the expression of *TraesCS2D02G274900.1* was up-regulated to a six-fold change on the 13th day under 25 °C and was decreased by a six-fold change between the 13th and 16th day. In addition, the expression of *TraesCS3B02G456800.1* also responded to the elevated temperature and its expression was specifically increased on the 13^th^ day at 20 °C. The expression of *TraesCS2D02G274900.1* and *TraesCS3B02G456800.1* in responding to the elevated temperature might have been due to the fact that they were both in the same phylogenetic group. Overall, although our work suggests that the relative expression levels of *TaCystatin* genes change greatly under stress conditions, their expression patterns under stress challenges were complex and varied.

## 3. Discussion

Cystatins in plants, as an intrinsic small protein, have been reported to play important roles in multiple stress-signaling pathways and are widely involved in the response to environmental stress [67,68,69]. Although several previous studies have identified and characterized cystatin members in *Arabidopsis,* rice, sorghum, barley, *Glycine max*, and *Brachypodium distachyon* [10,12,31,32], knowledge of the *cystatin* family of bread wheat has still been limited until now. As bread wheat (*Triticum aestivum*) occupies a significant position in the supply of food crops for humans, here, we identified and characterized 55 *TaCystatin* family members using the latest completion of the wheat genome [35]. According to the phylogenetic and gene structure analysis (Figure 1 and Figure 2a), 55 TaCystatins could be categorized into three groups (Groups A, B, and C), consistent with previous findings [6,30]. We found that the structural domains, gene structures, and motif compositions of the *TaCystatin* family were relatively conserved within each group (Figure 2b and Figure 6b,c). In addition, a majority of TaCystatins were predicted to be localized in the extracellular components (Table 1), which might be attributed to the fact that cystatins are reversible inhibitors of C1A and C13 proteinases, and most of the C1A and C13 proteinases in plants were localized in the extracellular components [9,70].

The number of cystatins in wheat was the highest among the several plant species (Table 2). This might be due to the fact that wheat had undergone two whole genome duplications and wheat (16 Gb genome size; genomes BBAADD) both is a complex allohexaploid and has a large genome (Table 2). To investigate the reasons for the high number of *cystatin* genes in wheat, the homoeologous groups for the *TaCystatin* family were analyzed. Approximately 43.65% of *TaCystatin* genes could be assigned to 1:1:1 homoeologous groups (Table 3), which was above the average homoeologous retention proportion in wheat (43.65% vs. 35.8%; Table 3) [35]. Moreover, we identified 11 and 25 orthologous gene pairs between *TaCystatins* and *cystatins* in *A. tauschii* and *T. dicoccoides* (Figure 4c and Table S4), respectively. Previous studies have demonstrated that *Aegilops tauschii* (genomes DD) is the natural source of D sub-genomes of wheat (genomes BBAADD) and that *Triticum dicoccoides* (BBAA) is the natural foundation of B and A sub-genomes of wheat (genomes BBAADD) [71]. Together, these results indicate that two whole genome duplications resulting from hybridization might partly be responsible for the abundance of cystatin members in wheat. However, even considering the ploidy level, we found that the number of *cystatin* genes in wheat was still more than three times that in rice (Figure S1). We found that in wheat, the proportion of *cystatin* genes with the homeology (1:1:N, N:1:1, or 1:N:1) was (14.55% vs. 5.7%; Table 3) higher than for all other wheat genes. This suggests that gene duplications play a vital role in the expansion of the *cystatin* family in wheat. Chromosomal locations and synteny analyses were then performed to explore the relationships within the *cystatin* genes family in wheat more comprehensively. The results showed that 55 *TaCystatins* were irregularly distributed on chromosome 1, 2, 3, 4, 5, and 7 (Figure S2), and the *TaCystatin* family members in the larger group were more likely to be in distal telomeric regions (Figure 3a and Table S2), which is also consistent with previous reports [36]. Tandem and segmental duplications have been reported to be the two main causes of gene duplication in plants [37]. In this study, eight tandem duplication clusters and forty-five collinear *cystatin* gene pairs were identified (Figure 3b), which suggests that both tandem and segmental duplication events were necessary for the expansion of the *cystatin* family in wheat, while segmental duplications appeared to be more advantageous in duplication patterns.

It is quite common for CUB to occur in the genome, which indicates that genes encoding proteins are not uniformly used. CUB is also considered to be important in gene regulation and molecular evolution [72,73]. To analyze the CUB of the *cystatin* family in plants, we calculated several representative parameters including CBI, Fop, ENC, GC3s, and GC contents in this study. Among them, G/C in the third base of the *cystatin* family was more preferable in monocotyledonous plants, whereas T/A in the third base of the *cystatin* family was more predominant in dicotyledonous plants (Figure 5a), which is consistent with most previous findings [46]. We also deciphered the relative synonymous codon usage (RSCU) of genes from the cystatin family in seven species. The results showed that the RSCU was relatively conserved with monocots and dicots, and the average RSCU of the *cystatin* family in monocots was higher than that in dicots (Figure 5d). In addition, the results of PR2 together with the neutral plot analysis showed that CUB of *cystatin* genes in both monocotyledonous and dicotyledonous plants were affected by mature pressure (Figure 5b,c, and Figures S3 and S4). Thus, the results showed that CUB of the genes from the *cystatin* family was relatively conserved in dicotyledonous or monocotyledonous plants, and the *cystatin* genes in monocotyledonous plants had enhanced codon preference compared to dicotyledonous plants.

*Cystatin* genes have shown divergent expression patterns in several plant species. For instance, the expression levels of *SbCys15* and *SbCys7* in *Sorghum bicolor* were considerably higher in vegetative tissues than in reproductive tissues, while others were more expressed in reproductive tissues [12]. In this study, we found that most of the selected *TaCystatin* genes were highly expressed in the top leaves (Figure 9) compared to that in RO, suggesting that they may participate in plant growth and development. We predicted cis-acting regulatory elements to analyze the putative biological functions of *TaCystatins* in the view of the promoter structure. The results showed that MeJA-responsive cis-acting and ABA-responsive cis-acting elements accounted for most among the predicted hormone-responsive elements. Additionally, both ABA and MeJA-responsive elements of predicted hormone-responsive elements were not absent in the promoter region of all members of the *TaCystatin* family (Figure 7). Thus, we analyzed the expression of *TaCystatins* upon ABA and MeJA application. The results suggested that the expression of *TaCystatins* was significantly changed under ABA or MeJA treatments (Figure 8). As MeJA and ABA have been reported to play important roles in response to different kinds of stress [58], we hypothesized that the *TaCystatin* family might be involved in the response to stress biology. Moreover, cystatins are thought to be widely used to regulate endogenous processes that respond to different kinds of abiotic or biotic stresses [68,74]. Therefore, we analyzed the expression of the *TaCystatin* family under biotic stress (viral infection) and abiotic stress (cold treatment). The results showed that the expression levels of the *TaCystatin* family changed significantly under biotic stress (viral infection) or abiotic stress (cold treatment) (Figure 10) and their relative expression levels varied significantly, which indicates that the *TaCystatins* might play specific roles under both viral infection and cold stress.

## 4. Materials and Methods

### 4.1. Identification of Cystatin Family in Wheat

The cystatin protein sequences of *Arabidopsis thaliana* and rice (*Oryza sativa*) were obtained from the Ensemble Plants database (http://plants.ensembl.org/index.html; updated on 12 February 2021) as previously described [10]. The cystatin protein sequences of *Glycine max*, barley (*Hordeum vulgare*), *Sorghum bicolor*, and *Brachypodium distachyon* were obtained from the Phytozome database (https://phytozome-next.jgi.doe.gov/info; accessed on 12 February 2021) as previously described [12,31,32]. The newly released reference genome of bread wheat (*Triticum aestivum*) used in this study was downloaded from the Ensemble Plants database (http://plants.ensembl.org/Triticum_aestivum/Info/Index; accessed on 12 February 2021). These cystatin sequences in *A. thaliana* and rice were used as queries to conduct local BlastP against the latest bread wheat genome (threshold E-value < 1 × 10^–10^). The hidden Markov model (HMM) profile (PF00031) of the *cystatin* family was downloaded from the PFAM database (http://pfam.xfam.org/; accessed on 13 February 2021). The cystatin HMM profile (PF00031) was used for functional annotation filters using the HMMER software (version 3.0) [75]. Then, all candidate protein sequences were further filtered using the NCBI Batch Web CD-Search Tool (https://www.ncbi.nlm.nih.gov/Structure/bwrpsb/Bwrpsb.cgi; accessed on 13 February 2021) to confirm the structural integrity of the cystatin domain in each tag sequence. In summary, 55 TaCystatins were identified. Detailed information on the *TaCystatin* family including gene locations, gene length, ORF length, and size were collected from the Ensemble Plants database. The theoretical PI, molecular weight (MW), and grand average of hydropathy (gravy) of TaCystatins were analyzed using the ExPAsy tool (https://web.expasy.org/compute; accessed on 13 February 2021) [76]. The subcellular localization of TaCystatins was predicted using the Plant-mPLoc tool [77].

### 4.2. Multiple Sequence Alignment and Phylogenetic Analysis

We conducted sequence alignment analysis of the cystatins from bread wheat, rice, *Arabidopsis*, and barley using MUSCLE in the MEGA X software (Mega Limited, Auckland, New Zealand) with default parameters. Then, all the sequences imported into the MEGA X software were used to construct a maximum likelihood (ML) phylogenetic tree, with a set of 1000 bootstrap replications and the Poisson distribution mode. We also used a similar method to build an ML phylogenetic tree of the TaCystatin protein sequences.

### 4.3. Gene Duplication Analysis of TaCystatins

McscanX software was used to investigate tandem and segmental duplications in the *TaCystatin* family [78]. The synteny relationships between several members of the *cystatin* family in bread wheat and several other species were analyzed using McscanX. Segmental and tandem duplication relationships were virtualized using the Advanced Cicros function of the TBtools software [50]. The Ka/Ks ratios were calculated for tandem duplications using the Ka/Ks Calculator function of the TBtools software and divergence times (T) were estimated on the basis of T = Ks/(2 × 9.1 × 10^−9^) Mya [38].

### 4.4. Codon Usage Pattern Analysis

The cystatin CDS sequences longer than 300 bp of bread wheat, *A. thaliana*, rice, barley, sorghum, *Glycine max*, and *Brachypodium distachyon* were obtained to calculate the parameters of codon usage bias using CodonW 1.4.2 software (http://codonw.sourceforge.net/; accessed on 16 March 2021) [79]. These parameters included GC content, GC3s content, frequency of optimal codons, and the codon bias index. GC12 content, relative synonymous codon usage (RSCU), and ENC were calculated using the EMBOSS tool (https://www.bioinformatics.nl/emboss-explorer/; accessedd on 16 March 2021).

### 4.5. Gene Structure and Motif Analysis

The genome feature format (GTF) file for bread wheat was downloaded from the Ensemble Plants database [80]. We used the gene structure view (advanced) function of the TBtools software to analyze and visualize the gene structures of *TaCystatins* [50]. We used motif-based sequence analysis tools (MEME) (https://meme-suite.org/meme/; accessed on 18 February 2021) to predict the conserved motifs of *TaCystatins*, with a set maximum section of up to 20 motifs [81]. Finally, the results were visualized using TBtools software [50].

### 4.6. Promoter Analysis

We extracted the 2000 bp upstream sequences of the transcription start site of genes from thd *TaCystatin* family using the GTF file of the bread wheat genome. The obtained sequences were submitted to the PlantCARE website (http://bioinformatics.psb.ugent.be/webtools/plantcare/html/; accessed on 21 March 2021) to predict the putative cis-acting regulatory elements.

### 4.7. Plant Cultivation and Viral Inoculation

Bread wheat (*T. aestivum* cv Yangmai 158) seedlings were cultivated in an artificial greenhouse at 23 °C with a 16/8 h (light/dark) photoperiod. Three-leaf-stage wheat seedlings were used for viral inoculation, temperature stress, and hormone treatments. CWMV and WYMV inoculations were performed by applying mechanical friction using in vitro synthesized transcripts, as previously described [82,83]. For temperature stress analysis, wheat seedlings were divided into four groups and laid up in greenhouses with different temperatures (8, 15, 20, and 25 °C), respectively. The plants placed under 8 °C were regarded as the mock control. For hormone treatments, methyl jasmonate (MeJA) and abscisic acid (ABA) were dissolved in 100% ethanol to suitable concentrations as stock solutions and then were diluted with sterile distilled water containing 0.1% Triton X-100. Wheat seedings (YangMai 158) were treated with 100 μmol L^−1^ MeJA and 100 μmol L^−1^ ABA, and 0.1% Triton X-100 was regarded as the mock control. Three biological replicates of leaf samples were collected at divergent times for RNA extraction and RT-qPCR analysis.

### 4.8. RNA Isolation and Real-Time Quantitative Polymerase Chain Reaction (RT-qPCR)

Total RNA was extracted using TRIzol Reagent (Invitrogen, Carlsbad, CA, USA). Then, strand cDNA was synthesized using a First Strand cDNA Synthesis Kit (TOYOBO, Kita-ku, Osaka, Japan). RT-qPCR was performed using an ABI QuantStudio5 Detection System (Applied Biosystems, Foster City, CA, USA) using the Hieff qPCR SYBR Green Master Mix (YEASEN, Shanghai, China). Each treatment was performed using at least three biological replicates, with three technical replicates. The relative expression levels of target genes were calculated in the 2^–ΔΔC(t)^ manner as described in a previous study [84]. The CDC gene was used as the internal reference for each reaction. The primers used for RT-qPCR are provided in Table S6.

## 5. Conclusions

In this study, we identified and characterized 55 members of the *cystatin* family in bread wheat, which could be divided into three phylogenetic groups. *TaCystatin* genes’ structure and the composition of the amino acid motifs in proteins were conserved in each of the three clades of this gene family. The homoeolog retention rate and gene duplication partly explain the expansion of this family and segmental duplications played a predominant role in duplication patterns. Codon usage pattern analysis showed that the *TaCystatin* family had an obvious codon preference. The expression of selected *TaCystatins* was organ-specific and greatly changed due to viral infection or cold stress, with several exceptions. Our results will be helpful to attain a comprehensive understanding of the *cystatin* family in wheat and to investigate the relationships between *TaCystatins* and responses to biotic or abiotic stress.

## Figures and Tables

**Table 1:** Detailed information of the putative cystatin proteins in bread wheat.

Sequence ID	Gene Location		ORF Length (bp)	Size (aa)	MW (KDa)	pI	GRAVY	Splice Variants	Subcellular Location
TraesCS2A02G576200.1	770342688	770343067	303	100	11.51618	9.07	−0.551	1	Cytoplasmic
TraesCS3D02G416600.1	528084470	528084787	318	105	11.42406	8.57	−0.016	1	Cytoplasmic
TraesCS4B02G067000.1	59866358	59866678	321	106	11.71911	6.83	−0.444	1	Mitochondrial
TraesCS1D02G310300.1	406552140	406552876	324	107	11.78746	6.74	−0.361	1	Cytoplasmic
TraesCS1B02G322100.1	546534514	546535277	324	107	11.77056	9.13	−0.293	1	Mitochondrial
TraesCS4D02G252400.1	421220747	421221148	342	113	12.32008	5.21	0.044	1	Extracellular
TraesCS7D02G337700.1	431120375	431120733	351	116	12.60957	9.4	0.039	1	Extracellular
TraesCS3B02G361700.1	573403587	573404657	354	117	12.74076	9.18	0.113	1	Extracellular
TraesCS7A02G341900.1	501921436	501921789	354	117	12.93808	9.3	0.126	1	Extracellular
TraesCS7B02G241000.1	448639521	448639874	354	117	12.83683	9.4	0.014	1	Extracellular
TraesCS4A02G052100.1	43031303	43031882	354	117	13.18154	10.11	−0.112	1	Mitochondrial
TraesCS5D02G502100.1	529800899	529801767	357	118	13.52646	5.01	−0.419	1	Cytoplasmic
TraesCS5A02G557600.1	708451918	708452506	357	118	12.96505	9.61	−0.057	1	Mitochondrial
TraesCS3D02G020500.1	6949696	6950286	357	118	12.49836	9.98	0.117	1	Extracellular
TraesCS4D02G066000.1	40824447	40824806	360	119	13.14997	9.15	−0.267	1	Extracellular
TraesCS4A02G247900.1	558676008	558676367	360	119	13.21215	9.15	−0.21	1	Mitochondrial
TraesCS2B02G148400.1	114430719	114431084	366	121	12.93972	9.59	−0.06	1	Mitochondrial
TraesCS4B02G396100.1	670456115	670456483	369	122	13.43764	9.76		1	Mitochondrial
TraesCS2A02G126100.1	74561376	74562320	372	123	13.01076	9.5	0.027	1	Extracellular
TraesCS2D02G128900.1	74985591	74986626	372	123	13.08292	10.12	−0.059	1	Extracellular
TraesCS3B02G077600.1	48667966	48668528	387	128	14.54467	5.88	−0.203	1	Cytoplasmic
TraesCS2D02G589700.1	645958642	645959221	387	128	14.58468	6.04	−0.241	1	Cytoplasmic
TraesCS2A02G578500.1	771772538	771773124	387	128	14.5586	6.04	−0.282	1	Cytoplasmic
TraesCS2B02G609300.1	789563953	789564540	387	128	14.47153	6.06	−0.178	1	Cytoplasmic
TraesCS2A02G581500.1	773562880	773563385	387	128	14.59085	6.4	−0.054	1	Extracellular
TraesCS2B02G611300.1	790623664	790624261	387	128	14.48565	6.83	−0.135	1	Chloroplast
TraesCS2D02G586800.1	644923731	644924419	387	128	14.48569	7.78	−0.141	1	Extracellular
TraesCS2B02G502800.1	697398239	697398802	387	128	14.58693	7.8	−0.163	1	Cytoplasmic
TraesCS2B02G611200.1	790612843	790613405	387	128	14.4467	8.93	−0.16	1	Extracellular
TraesCS2A02G576100.1	770290351	770290922	387	128	14.45468	8.95	−0.169	1	Extracellular
TraesCS2D02G586900.1	644975269	644975655	387	128	14.51576	9.14	−0.212	1	Extracellular
TraesCS2A02G575600.1	770023125	770023511	387	128	14.39963	9.26	0.005	1	Extracellular
TraesCS2D02G477600.1	579510589	579511163	387	128	14.55305	9.33	−0.109	1	Cytoplasmic
TraesCS3B02G456600.1	698000195	698000638	393	130	13.82574	7.67	0.068	1	Chloroplast
TraesCS3D02G325100.1	437966538	437967959	429	142	15.71427	6.14	−0.056	1	Cytoplasmic
TraesCS5D02G502600.1	529845043	529846275	429	142	15.68523	8.8	−0.136	1	Extracellular
TraesCS5A02G487700.1	657756833	657758161	429	142	16.08189	9.69	−0.142	1	Mitochondrial
TraesCS5D02G502200.1	529815663	529817000	435	144	16.3071	7.95	−0.092	1	Extracellular
TraesCS2A02G275900.1	455481094	455481712	435	144	15.05153	10.01	0.183	1	Chloroplast
TraesCS2B02G293600.1	409400473	409401171	435	144	14.78817	10.16	0.202	1	Chloroplast
TraesCS2D02G274900.1	344303051	344303637	435	144	14.93736	10.23	0.166	1	Chloroplast
TraesCS3B02G456800.1	698194875	698195312	438	145	15.55955	8.55	−0.24	1	Extracellular
TraesCS3A02G421000.1	662520087	662520865	456	151	16.17131	7.66	−0.164	1	Extracellular
TraesCS4D02G025600.1	10999710	11000093	384	127	13.30729	9.17	0.227	1	Extracellular
TraesCS3B02G456700.1	698122257	698122715	459	152	16.27537	7.66	−0.199	1	Extracellular
TraesCS3D02G416700.1	528094859	528095317	459	152	16.10226	7.66	−0.114	1	Extracellular
TraesCS3D02G416500.1	527735678	527736139	462	153	16.20031	8.43	−0.15	1	Extracellular
TraesCS3B02G456500.1	697983253	697983714	462	153	16.19928	8.45	−0.112	1	Extracellular
TraesCS5A02G487600.1	657725166	657725966	507	168	19.1852	6.16	−0.283	1	Extracellular
TraesCS1D02G255800.1	348063436	348067242	513	170	18.15371	9.42	−0.115	1	Extracellular
TraesCS1B02G267100.1	469999341	470003147	543	180	19.01667	7.79	−0.018	1	Extracellular
TraesCS1A02G256400.1	448926775	448930744	546	181	19.11479	8.62	−0.044	2	Chloroplast
TraesCS3D02G189800.1	177482785	177486000	732	243	26.60537	6.37	−0.249	1	Extracellular
TraesCS3A02G185800.1	218117459	218120388	732	243	26.60537	6.37	−0.249	1	Extracellular
TraesCS3B02G215400.1	256357151	256360095	732	243	26.74851	6.38	−0.288	1	Extracellular
TraesCS3B02G215400.1	256357151	256360095	732	243	26.74851	6.38	−0.288	1	Extracellular

**Table 2:** Number of cystatin proteins in seven different species.

Lineage	Organism	Genome Size	Total Number of Cystatin Proteins
**Dicots**	*Arabidopsis thaliana* (2n)	135 Mb	7
	*Glycine max* (2n)	1.15 Gb	20
**Monocots**	*Oryza sativa* (2n)	500 Mb	11
	*Sorghum bicolor* (2n)	700 Mb	22
	*Hordeum vulgare* (6n)	1.35 Gb	13
	*Brachypodium distachyon* (2n)	300 Mb	23
	*Triticum aestivum* (6n)	15.8 Gb	55

**Table 3:** Groups of homeologous *cystatin genes* in bread wheat.

Homoeologous (A:B:D)	All Wheat Genes ^1^	Classes ^2^			Number of Groups	Number of Genes	% of Total TaCystatins
		A	B	C			
1:1:1	35.8%	2	1	5	8	24	43.65%
n: 1:1, 1: n:1, or 1:1: n ^&^	5.7%		1	1	2	8	14.55%
1:1:0, 1:0:1, or 0:1:1	13.2%	2		2	4	8	14.55%
Orphans	37.1%			1	1	1	1.81%
Other ratios	8%	1	1	2	4	14	25.45%

^&^ n > 1. ^1^ All wheat genes distributed among homeologous groups of the whole wheat genome according to IWGSC. ^2^ The number of cystatin family members within each phylogenetic group (A, B, and C).

**Table 4:** Codon usage indicators of the *cystatin* family in seven different species. Abbreviations: CBI, codon bias index; Fop, frequency of optimal codons; ENC, effective number of codon; and GC3s, contents of G or C bases at the third position of the codons; and GC content, the contents of the G and C bases of the codons.

Species Name	CBI	Fop	ENC	GC3s	GC Content
*Triticum aestivum*	0.093	0.470	42.185	0.774	0.611
*Arabidopsis thaliana*	0.010	0.423	52.233	0.509	0.473
*Brachypodium distachyon*	0.109	0.479	40.929	0.810	0.626
*Hordeum vulgare*	0.096	0.474	41.722	0.770	0.604
*Glycine max*	0.028	0.433	50.735	0.595	0.508
*Oryza sativa*	0.106	0.477	38.076	0.857	0.669
*Sorghum bicolor*	0.094	0.468	42.536	0.794	0.634

## Data Availability

Not applicable.

## Supplementary Materials

Supplementary materials can be found at https://www.mdpi.com/article/10.3390/ijms221910264/s1.
