# Supplementary figures and images for: Genome-Wide Identification and Characterization of the Cystatin Gene Family in Bread Wheat (Triticum aestivum L.)
Source: Int J Mol Sci. 2021 Sep 23;22(19):10264. doi: 10.3390/ijms221910264 (PMC8508539; doi:10.3390/ijms221910264)

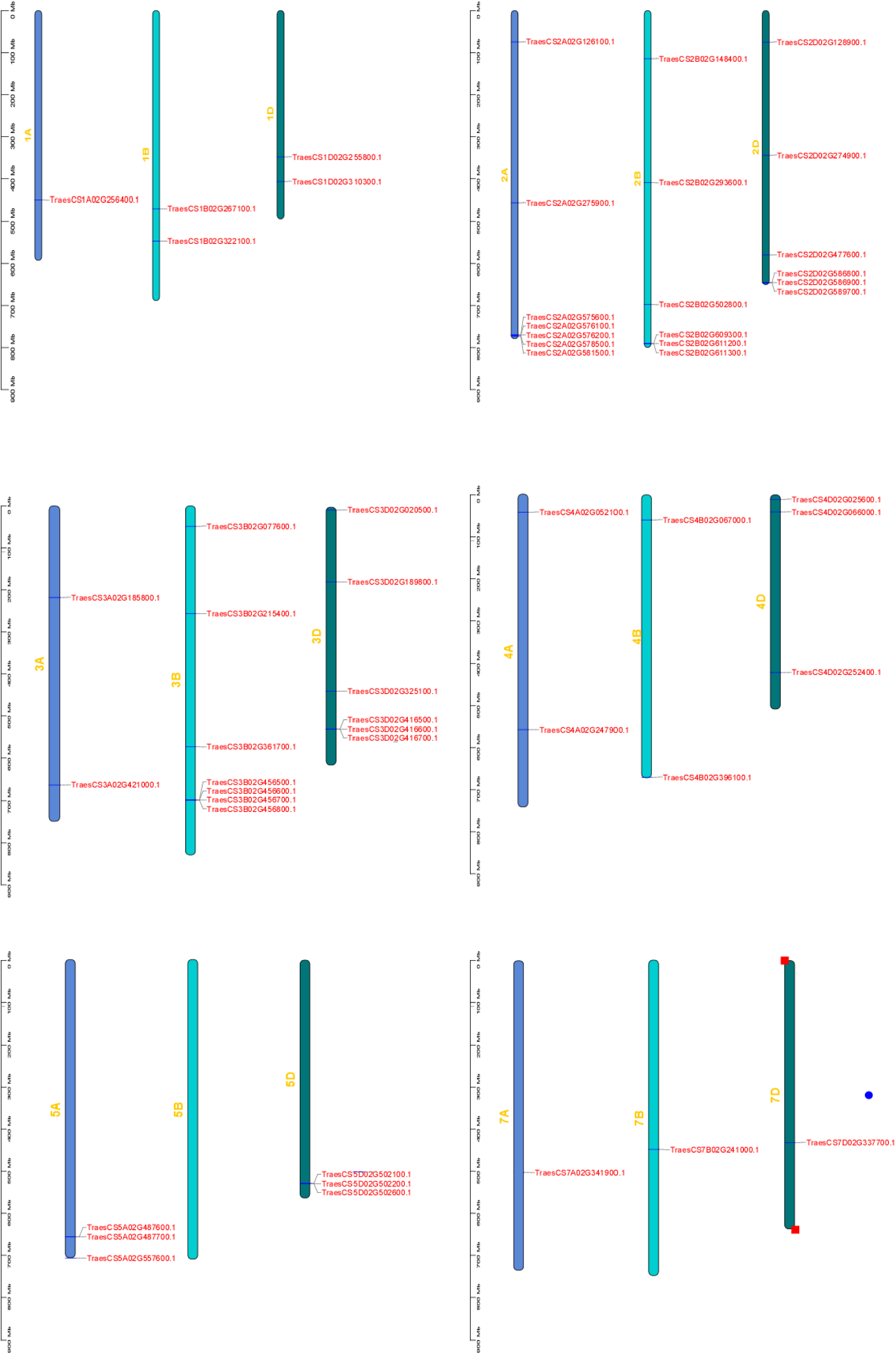

Supplement: Supplementary file 1 [file ijms-22-10264-s001.zip › Figure S2.tif]

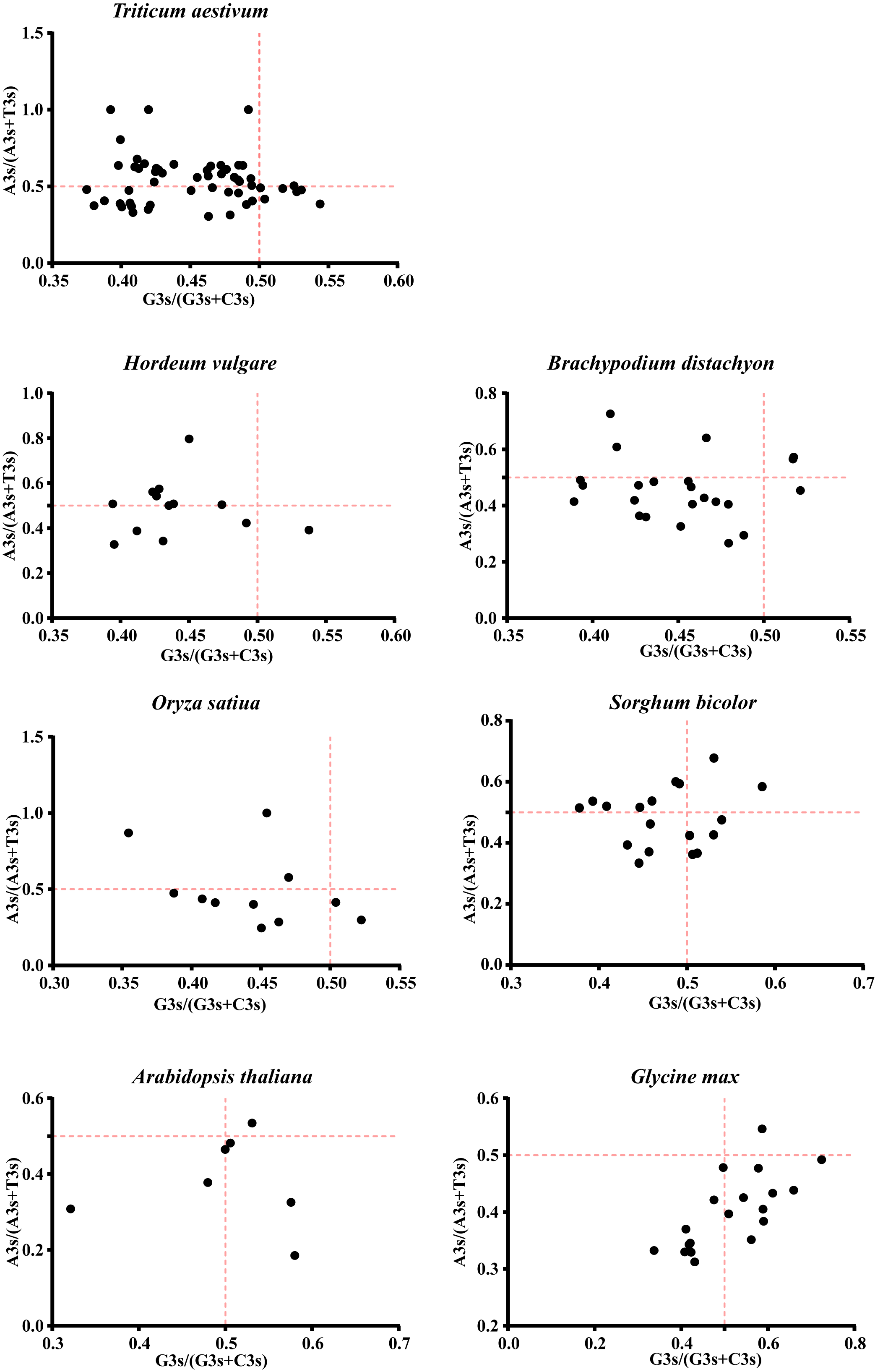

Supplement: Supplementary file 1 [file ijms-22-10264-s001.zip › Figure S3.tif]

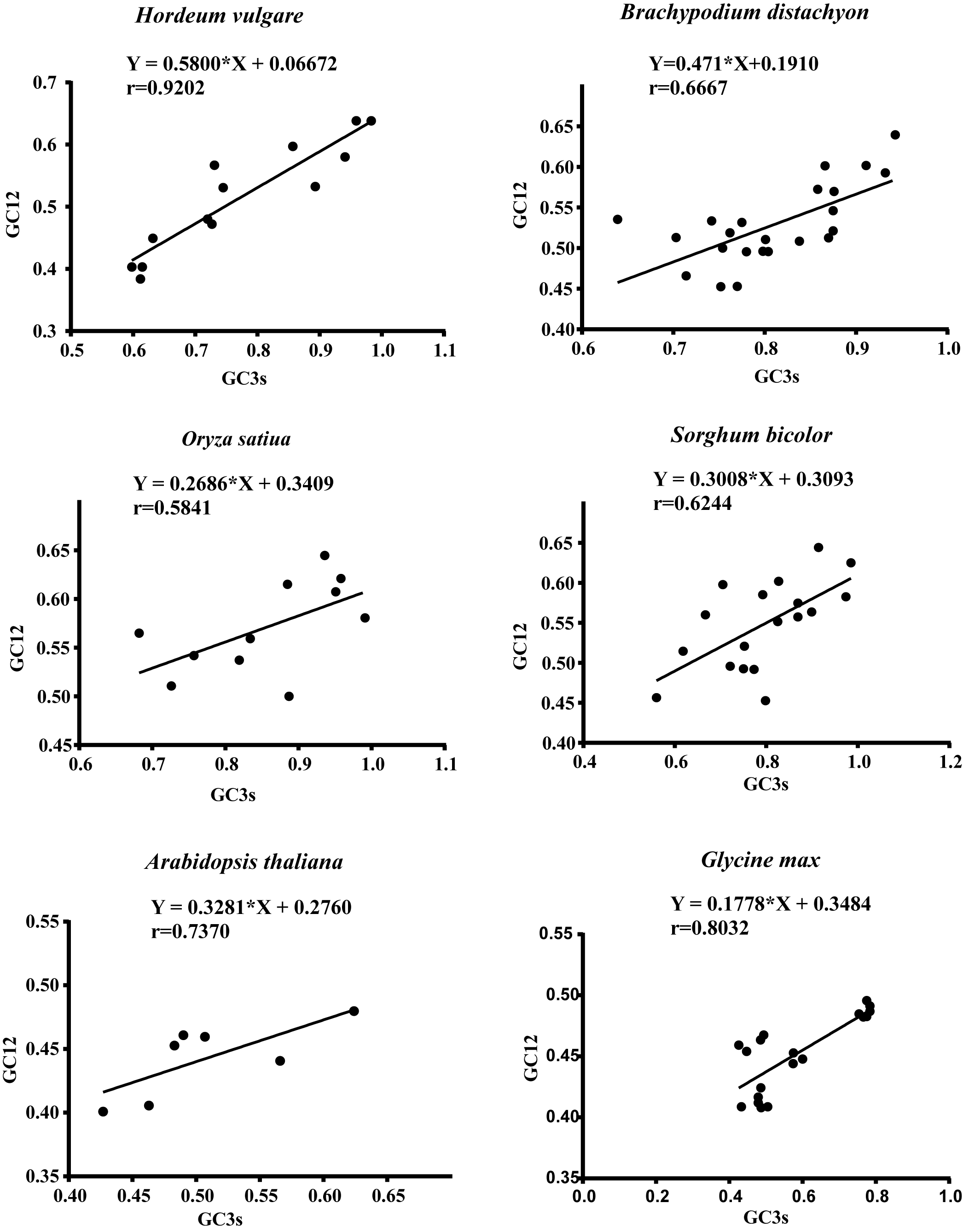

Supplement: Supplementary file 1 [file ijms-22-10264-s001.zip › Figure S4.tif]

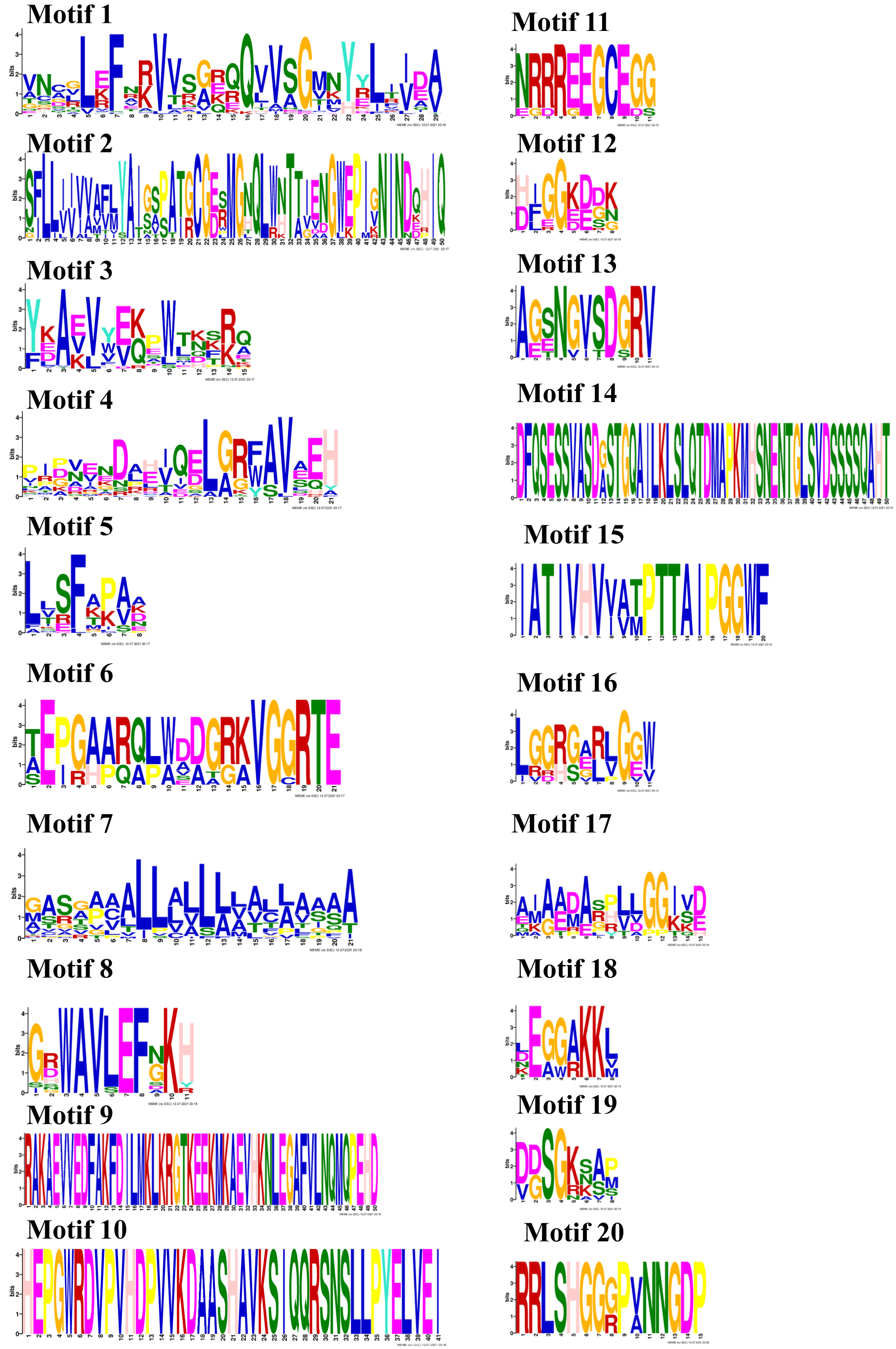

Supplement: Supplementary file 1 [file ijms-22-10264-s001.zip › Figure S5.tif]
